# The National Emergency Medical Service Role During the COVID-19 Pandemic in Sierra Leone

**DOI:** 10.1017/S1049023X20001211

**Published:** 2020-09-22

**Authors:** Marta Caviglia, Riccardo Buson, Sara Pini, Amara Jambai, Matthew Jusu Vandy, Francesco Venturini, Paolo Rosi, Francesco Barone-Adesi, Francesco Della Corte, Luca Ragazzoni, Giovanni Putoto

**Affiliations:** 1. CRIMEDIM – Research Center in Emergency and Disaster Medicine, Università del Piemonte Orientale, Novara, Italy; 2. Doctors with Africa – CUAMM, Padova, Italy; 3. Ministry of Health and Sanitation, Freetown, Western Area, Sierra Leone; 4. Venice Emergency Service (SUEM 118), Venice, Italy

**Keywords:** COVID-19, Emergency Medical Services, prehospital care, Sierra Leone, CCC, community care center, CMP, case management pillar, COVID-19, novel coronavirus disease 2019, CRIMEDIM, Research Center in Emergency and Disaster Medicine, CTC, COVID-19 treatment center, EOC, emergency operations center, IPC, infection prevention and control, MoHS, Ministry of Health and Sanitation, NEMS, National Emergency Medical Service, OC, operation center, PPE, personal protective equipment, SOP, standard operating procedure

## Abstract

This report describes the main adaptive and transformative changes adopted by the brand-new National Emergency Medical Service (NEMS) to face the novel coronavirus disease 2019 (COVID-19) in Sierra Leone, including ambulance re-distribution, improvements in communication flow, implementation of ad-hoc procedures and trainings, and budget re-allocation. In a time-span of four months, 1,170 COVID-19 cases have been handled by the NEMS through a parallel referral system, while efforts have been made to manage the routine emergencies of the country, causing a substantial intensification of daily activities.

**Event Identifiers**
1. a. Event Type: Pandemic
2. b. Event Onset Date: March 31, 2020
3. c. Location of Event: Sierra Leone
4. d. Dates of Observations Reported: March 31, 2020 - July 31, 2020
5. e. Response Type: Pandemic Management and Emergency Response

## Introduction

Sierra Leone reported the first confirmed novel coronavirus disease 2019 (COVID-19) case on March 31, 2020.^1^ By the end of July, the country had recorded a total number of 1,823 confirmed cases and 67 total deaths, thus rapidly changing the transmission pattern of the disease from sporadic cases to clusters and community transmission.^2,3^

Starting from October 2018, Sierra Leone has implemented the National Emergency Medical Service (NEMS), a coordinated prehospital referral system managed by Doctors with Africa (CUAMM; Padova, Italy) in collaboration with Veneto Region and Research Center in Emergency and Disaster Medicine (CRIMEDIM; Università del Piemonte Orientale; Novara, Italy), under the direct supervision of the Ministry of Health and Sanitation (MoHS; Freetown, Western Area, Sierra Leone). Since the beginning of the COVID-19 pandemic, the NEMS has been actively engaged in the national preparedness response plan to ensure a resilient referral system, able to effectively continue delivering routine services, and at the same time manage the sudden demands of referral of COVID-19-related cases. To fulfil this aim, NEMS’ efforts have been directed towards the implementation of absorptive, adaptive, and transformative capacities representing the three levels of resilience of a health system when exposed to a shock, as based on the Dimensions of Resilience Governance framework.^4^

## Data and Information Source

This field report is based upon NEMS monthly financial and management internal reports as well as on data collected though the NEMS operation center (OC) software, data shared by the MoHS, and by key stakeholders.

## Observations

Learning form the Ebola experience (2014-2016) and thanks to funds received from the World Bank (Washington, DC USA) and the United Nations (New York USA), in April 2020, the Government had promptly implemented a national preparedness and response plan that featured different elements, including the set-up of COVID-19 treatment centers (CTCs) and community care centers (CCCs).^5^ The response has been strengthened under the coordination of an emergency operations center (EOC) and was supported by several public health measures and recommendations such as the closing of public spaces, travel restrictions, physical distancing, and improved hygiene practices.^5,6^

The NEMS management team has been integrated in the case management pillar (CMP), a dedicated team of experts composed by members of the MoHS, the Republic of Sierra Leone Army Force, key partners, and stakeholders operating in the health care sector. The prime responsibility of the CMP was to devise and implement protocols and procedures for the management and referral of COVID-19 suspect, probable, and confirmed cases on a national scale. In line with the international guidelines, the CMP has agreed to comply to the case definition as showed in Table 1, according to clinical, epidemiological, and laboratory criteria.^3^ The role of the NEMS in supporting the national response to COVID-19 in Sierra Leone was two-fold. First and foremost, NEMS ambulance teams were in charge of transporting suspect, probable, or confirmed cases to referral health care facilities following the standard operating procedures (SOPs) defined by the CMP. Secondly, a parallel referral system was set up for the transport of COVID-19 specimens to the different laboratories.

**Table 1.** Case Definition for Novel Coronavirus Disease 2019 (COVID-19) Adopted in Sierra Leone During the National Response to the Pandemic

Suspect	Acute respiratory infection associated with fever or history of fever >38°C, shortness of breath, cough or sore throat, who has had either close contact with a confirmed or probable case of COVID-19 or has visited/stayed in a country with community transmission of COVID-19 in the 14 days prior to onset of the symptoms.
Probable	A suspect case for whom laboratory testing for COVID-19 is inconclusive (inadequate samples or indeterminate results).
Confirmed	Laboratory confirmation COVID-19 infection, irrespective of clinical signs and symptoms.

The NEMS internal and external communications underwent adaptive and transformative changes that altered the structure of information flow. The main goal of the CMP was to effectively coordinate all the actors involved in the response by creating direct bridges between them, while allowing NEMS to maintain the same control on its communication structure. The NEMS OC normally functions as main link between the caller requesting emergency medical assistance, represented by a health worker responsible for primary assessment of patients in a peripheral health unit, and the NEMS emergency care resources. To facilitate information flow in the COVID-19 response, the OC in-house software was upgraded to ease the triage process of COVID-19 emergency calls and to appropriately report the transportation of the COVID-19 patients. Specifically, in the event of a suspected or confirmed case notified by the EOC, the software allowed the OC operator access to a specific COVID-19 section and to mark signs, symptoms, and epidemiological data as appropriate, choosing between fever, cough, sore throat, difficulties in breathing, history of travel, and contact with confirmed cases. In a second step, the triage process was finalized with the communication of a patient’s vitals by the NEMS paramedic on scene. A “red code” was identified when either body temperature was >38°C, respiratory rate >20, systolic blood pressure <100, peripheral capillary oxygen saturation (SpO2) <94%, or heart rate >100.

**Figure 1.**
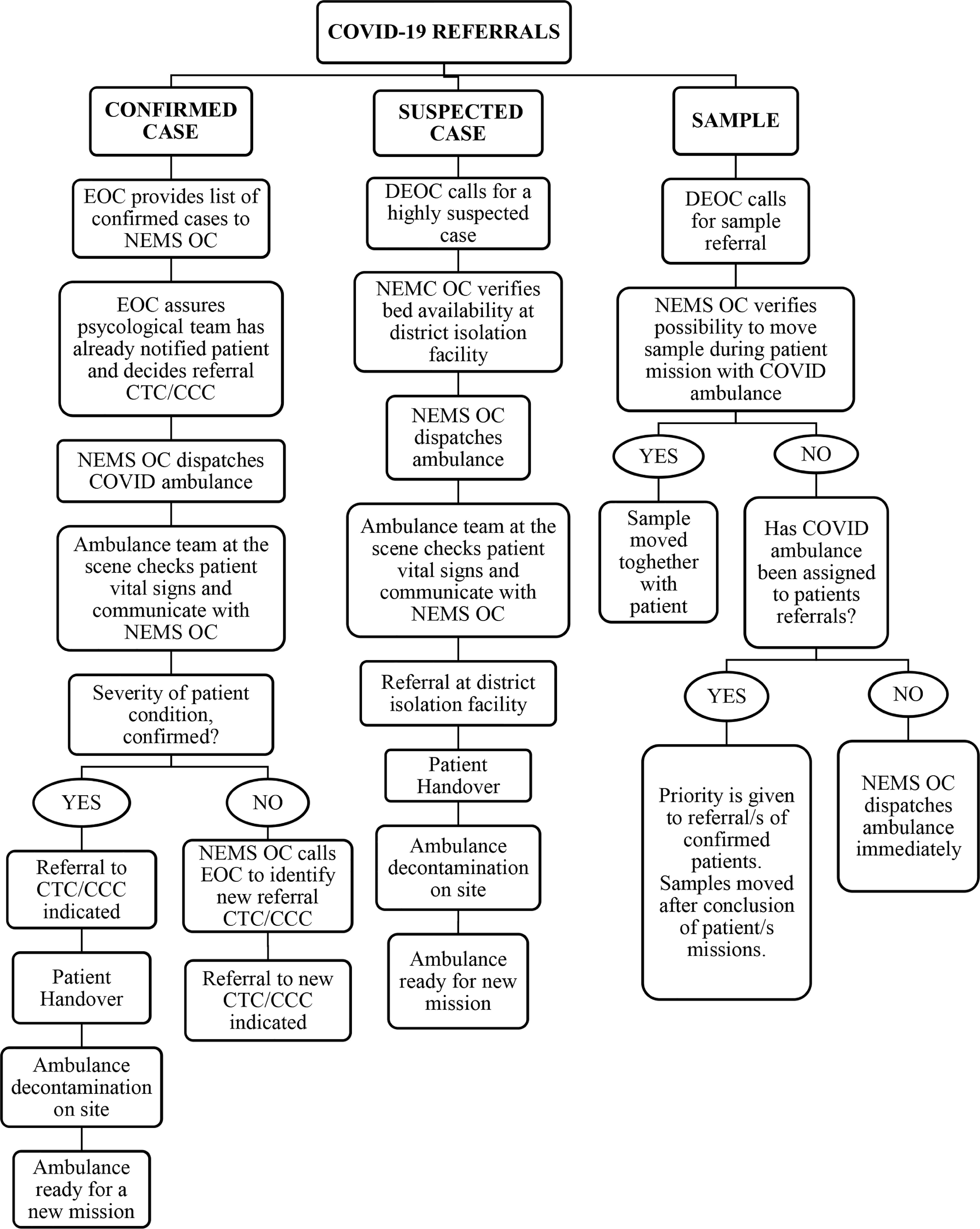
Communication Flow between the NEMS OC and Other Actors Involved in the Management and Referral of COVID-19 Cases. Abbreviations: NEMS, National Emergency Medical Services; OC, operation center.

Moreover, the public was encouraged to call the 117 special emergency number, previously used by private citizens for any Ebola-related concerns, to report COVID-19 symptoms directly to the NEMS OC. The district EOC in charge of situation assessment and patient management provided instruction to the OC team leaders on both ambulance dispatch and final destination to the designated referral hospitals, represented either by CTCs for severe cases, CCCs, or district hospitals equipped with isolation units in cases of mild disease or asymptomatic patients. Bed availability in the abovementioned centers was communicated daily to the NEMS OC. Figure 1 shows the diagram of communication flow between the NEMS OC and the other actors involved in the management and referral of the COVID-19 cases.

**Figure 2.**
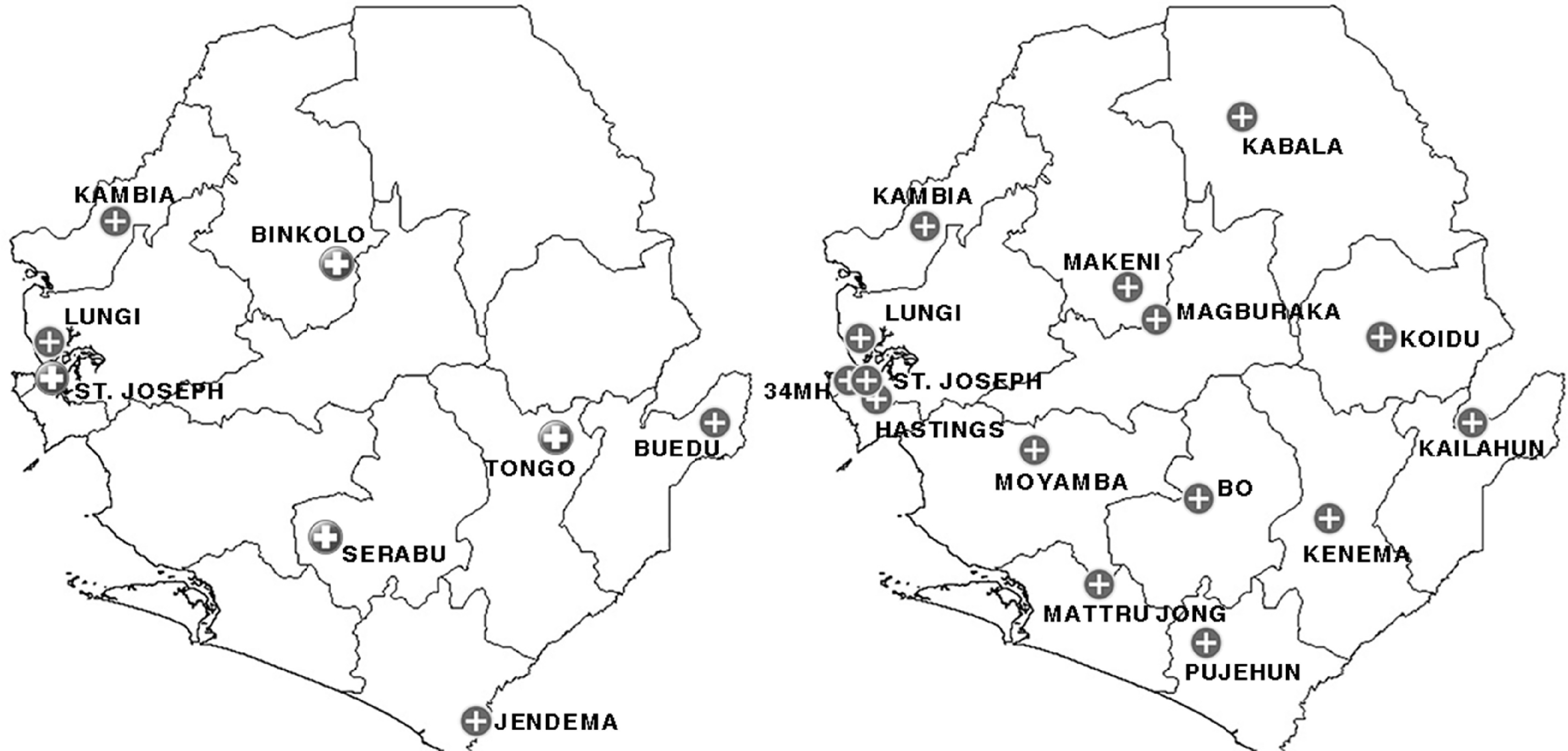
Location of Ambulances Allocated Specifically for the COVID-19 Response.

Ambulance distribution criteria entailed some organizational adaptations to effectively respond to the changing environment caused by the pandemic, using the same level of resources and capacities available in the country. In a first planning phase characterized only by sporadic cases locally detected, eight out of 81 ambulances had been allocated specifically for the COVID-19 response. Four of these ambulances had been positioned in all the principal points-of-entry represented by land access and the Lungi International Airport; the other four had been distributed on a regional basis (Figure 2). As the transmission pattern of the disease changed to cluster of cases and community transmission, the number of dedicated ambulances was increased to one per district, reaching the total number of 15. Three of these ambulances had been located in the more densely populated Western Area (Figure 2). The abovementioned ambulances had been selected according to the geographical area covered so to guarantee an acceptable response time, utilization rate, and geographical distribution of the other 66 ambulances used for routine emergencies.

Several SOPs were implemented to safeguard both the NEMS ambulance teams, composed by a trained paramedic and an ambulance driver, and the patients. To this end, the provision of an adequate number of personal protective equipment (PPE) was guaranteed by the NEMS procurement office well in advance by diverting 25% of NEMS monthly budget to this purpose. Ambulance teams were provided with full PPE, including FPP3 masks, gowns, gloves, goggles, and were instructed to have patients always wearing a surgical mask. Relatives of the patients were not allowed in the ambulance during transport to the health care facility. At the end of every mission, the ambulance remained inoperative for the time needed to clean all the equipment and manage the waste following the proper infection prevention and control (IPC) procedures. The District Ambulance Supervisor (DAS) oversaw the work of ambulance teams and ensured that they complied with all the procedures abovementioned.

As part of the national preparedness and response plan, different types of training sessions have been delivered to NEMS personnel, under the coordination and supervision of the CRIMEDIM. As Italy has been one of the most affected countries by the COVID-19 pandemic, researchers from CRIMEDIM have developed an ad-hoc training by integrating the Italian experience with the Sierra Leone national regulations and SOPs.^7^ A COVID-19 Special Training has been provided initially to the 98 paramedics and ambulance drivers identified for the management of COVID-19 cases. Subsequently, the training has been delivered to all the remaining ambulance teams. It consisted of a one-day training that included both theoretical and practical sessions focused on the correct use of PPEs and IPC procedures. In parallel, the 30 OC operators have participated in a one-day training on COVID-19 case definition, triage, and dispatch procedures. Of note, a low absenteeism rate was reported amongst the NEMS workforce. This entails both a strong commitment of the NEMS personnel, even in time of difficulties and personal danger, and the existence of effective strategies to improve their engagement. On the contrary, health care facilities in the country are reporting high levels of absenteeism due to fear of contagion, similarly to what happened during the Ebola epidemic.^8^

## Analysis

**Figure 3.**
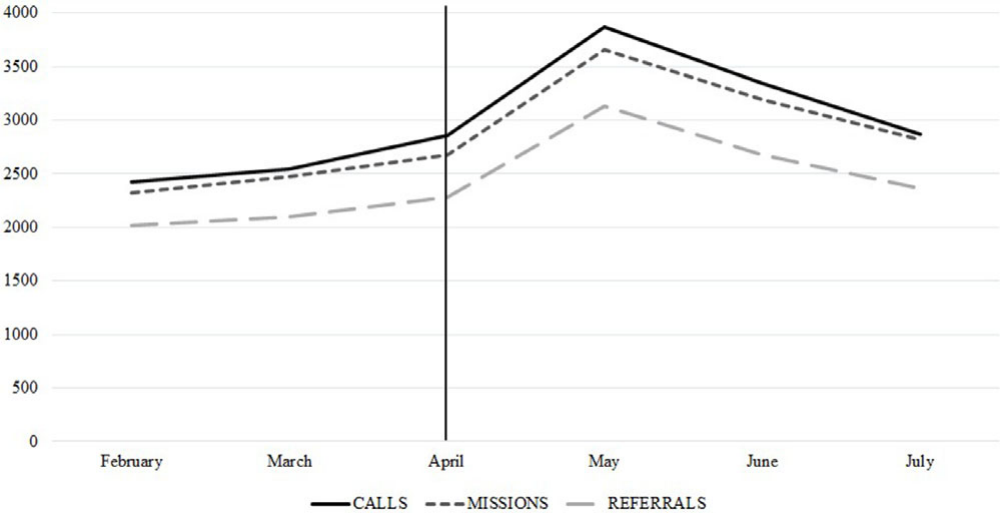
Upward Trend in NEMS Activities, Determined by the COVID-19 Outbreak.

The COVID-19 outbreak determined an upward trend in NEMS activities (Figure 3). According to the NEMS OC database, from April 2020 through July 2020, a total of 1,033 confirmed cases and 137 probable or suspected cases have been referred by NEMS to the pre-identified treatment centers or isolation units. In the same period, a total of 257 missions were related to transportations of COVID-19 patients. The number of confirmed cases referred accounts for the 64% of the total confirmed cases in the country in the abovementioned timeframe. This figure acquires considerable importance when considering that NEMS referrals covered the entirety of long-distance transports, whereas the remaining 36% of confirmed cases in the country were identified in urban areas where hospital facilities are either at walking distance or easily accessible through short-distance transports.

From a financial point of view, the COVID-19 outbreak had a significant impact on NEMS. As expected, the need of PPEs, ambulance decontamination kits, and special equipment such as digital thermometers increased dramatically, demanding timely reallocation of the existing budget. Furthermore, the ambulance fleet reduced by 15 had to cover routine emergencies in a much larger area, thus increasing costs related to gasoline and wear costs of the vehicles. Lastly, the supplementary training sessions delivered to all the NEMS personnel required movement of additional funds. The NEMS involvement in the national response plan was not exempt from challenges. First of all, stigma and fear were still associated with ambulances years after the devastating Ebola epidemic, especially in the most rural areas of Sierra Leone.^9^ This perception of fear and mistrust in the health care system recurred to some extent during the COVID-19 pandemic, with NEMS ambulance teams experiencing multiple cases of violence, including acts of vandalism against ambulances and refusal to be transported to treatment centers. To address the issue, the involvement of a psychological team in charge of adequately informing the patient and relatives about their condition and the need of isolation played a major role and showed some encouraging results.

The connection between the public emergency number 117 and NEMS OC established for the COVID-19 response has brought indirect consequences on the whole system. The route of emergency calls to NEMS OC through 117 has indeed not only been limited to coronavirus cases, but widened to all the typology of emergencies, resulting in an official opening of the NEMS service to the public. While the implementation of a public emergency number was part of the original design of NEMS, its abrupt activation during the current crisis has notably increased the number of incoming calls to the OC, testing the copying capacity of the whole system. Although the situation in Sierra Leone remains extremely challenging due to the vulnerability of its health sector, efforts to mitigate the impact of the pandemic and to implement rapid and effective response measures have received a considerable boost from the presence of a structured and fully equipped prehospital service.
